# From intensive care monitors to cloud environments: a structured data pipeline for advanced clinical decision support

**DOI:** 10.1016/j.ebiom.2024.105529

**Published:** 2024-12-27

**Authors:** Sijm H. Noteboom, Eline Kho, Maria Galanty, Clara I. Sánchez, Frans C.P. ten Bookum, Denise P. Veelo, Alexander P.J. Vlaar, Björn J.P. van der Ster

**Affiliations:** a Department of Anaesthesiology, Amsterdam UMC, University of Amsterdam, Meibergdreef 9, 1105 AZ, Amsterdam, the Netherlands; b Department of Intensive Care, Amsterdam UMC, University of Amsterdam, Meibergdreef 9, 1105 AZ, Amsterdam, the Netherlands; c Informatics Institute, University of Amsterdam, Meibergdreef 9, 1105 AZ, Amsterdam, the Netherlands; d Data Management, Amsterdam UMC, Meibergdreef 9, 1105 AZ, Amsterdam, the Netherlands; e Laboratory of Experimental Intensive Care and Anaesthesiology, Amsterdam UMC, University of Amsterdam, Meibergdreef 9, 1105 AZ, Amsterdam, the Netherlands

**Keywords:** Cloud environments, Data-driven algorithms, Data management, Real-time decision-making

## Abstract

**Background:**

Clinical decision-making is increasingly shifting towards data-driven approaches and requires large databases to develop state-of-the-art algorithms for diagnosing, detecting and predicting diseases. The intensive care unit (ICU), a data-rich setting, faces challenges with high-frequency, unstructured monitor data. Here, we showcase a successful example of a data pipeline to efficiently move patient data to the cloud environment for structured storage. This supports individual patient analysis, enables largescale retrospective research, and the development of data-driven algorithms.

**Methods:**

Since June 2021, ICU data of the Amsterdam UMC have been collected and stored in a third-party cloud environment which is hosted on large virtual servers. The feasibility of the pipeline will be demonstrated with the available data through research and clinical use cases. Furthermore, privacy, safety, data quality, and environmental impact are carefully considered in the cloud storage transition.

**Findings:**

Over two years, data from over 9000 patients have been stored in the cloud. The availability, agility, computational power, high uptime, and streaming data pipelines allow for large retrospective analyses as well as the opportunity to implement real-time prediction of critical events with machine learning algorithms. Critical events can be accessed by applying keyword search in the natural language data, annotated by the treating team. Besides, the cloud environment offers storage of institutional data enabling evaluation of healthcare.

**Interpretation:**

The combined data and features of cloud environments offer support for predictive algorithm development and implementation, healthcare evaluation, and improved individual patient care.

**Funding:**

University of Amsterdam Research Priority Agenda Program AI for Heath Decision-Making.

**Research in context**

**Evidence before this study**

The increasing demand for storage and handling of data in healthcare requires a solid management plan. Since healthcare handles sensitive personal information, privacy, confidentiality and security are paramount. Cloud environments offer a solution, since they not only offer storage capacity and computational power, but also offer strong security measures. Industries like finance and government, where handling sensitive personal information is standard daily practice, cloud environments have proven to be both secure and reliable.

**Added value of this study**

This study demonstrated the successful implementation of a cloud-based data management pipeline in the ICU of Amsterdam UMC. Over a period of two years, data from more than 9000 patients were collected and stored in a cloud environment, showcasing the feasibility and advantages of this approach. Key contributions include scalability to manage large data volumes, integration of diverse ICU data types, including high-frequency timeseries and natural language data from the electronical medical records. Furthermore, this study highlights the implementation of strict privacy and security measures compliant with GDPR.

Moreover, the study highlights the real-time data processing capabilities of the cloud environment, supporting both retrospective analyses and real-time predictive analytics for critical event prediction and proactive patient care.

**Implications of all the available evidence**

The findings demonstrate that cloud-based data management systems enhance real-time and retrospective data analysis in ICU settings and supporting predictive algorithms for improved patient care. Robust privacy and security measures ensure safe implementation. Future research should validate these benefits, explore automated event registration, and expand usage across departments. This study exemplifies the advantages and feasibility of adopting cloud-based systems in healthcare, encouraging broader adoption and innovation in data-driven clinical decision-making.

## Introduction

Continuous monitoring of vital parameters and machine settings contributes to the timely adjustment of treatment and support settings, enhancing understanding of complex cases in the intensive care unit (ICU).^1^ Advances in technological development, particularly exchange and usage of data, play an important role in intensive care medicine.^2^^,^^3^ Large intensive care databases, containing high-frequency monitor data, have the potential to identify trends, predict complications, and suggest treatment options.^4^ Additionally, integrating these data with electronic medical record (EMR) systems could provide a solid foundation for further understanding and improvement of intensive care.^5^ The stored data enable retrospective analysis, providing insight into diagnosis, progression, and underlying (patho)physiology of disease. Besides improving daily individualised clinical practice, such data also contribute to training algorithms to predict deterioration of patients and support clinical decision-making, ultimately leading to more personalised and effective care plans.^6^, ^7^, ^8^

However, such data, especially high-frequency continuous signals, require substantial storage space. Therefore, a concrete data management pipeline is required to store and facilitate optimal use of the obtained data. Structured data management, including sufficient data infrastructure, is often lacking and remains a barrier for the optimal use of data in research as well as in clinical practice.

In this paper, we will showcase a structured data management pipeline, using cloud server-based data warehousing. Data collected in the ICU will be stored and structured in a cloud-based data warehouse that is able to effectively address the limitations and challenges faced by traditional on-premise data warehouses in terms of scalability, cost efficiency, performance, and agility.^9^ The feasibility of the presented pipeline will be assessed by giving an overview of all available data and use cases in the Amsterdam University Medical Centre (Amsterdam UMC), Amsterdam, The Netherlands. Additionally, important challenges will be discussed in terms of privacy, safety, legal issues, and environmental impact.

## Methods

**Fig. 1:**
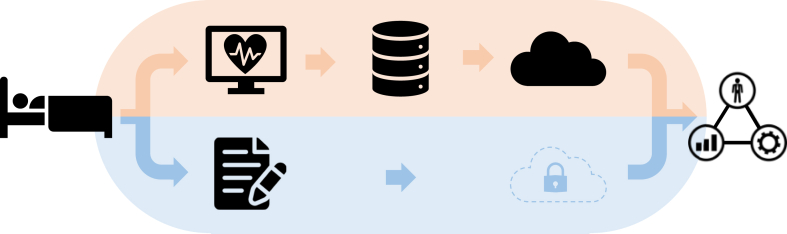
**Graphic representation of the data pipeline in the ICU of the Amsterdam UMC; from ICU patient to end-user. High-frequency timeseries (monitor) data from the ICU bed is collected in the local data warehouse. The monitor data is subsequently assembled in the cloud environment. The patient information data is directly collected from the EMR and stored into the cloud environment and is pseudonymised by default**.

In the Amsterdam UMC, we developed and implemented a data pipeline linking data collection in the ICU to controlled end-user access, see Fig. 1. Raw data from various sources in the ICU serves as the input of the pipeline. In the pipeline, an on-premise data warehouse enables storage and integration of vast amounts of data from various sources.^10^ Subsequently, the data are transmitted into the cloud-based data warehouse, from where the end-user can query data for retrospective analysis.

### Data at the ICU

Since June 2021, the ICU of the Amsterdam UMC, has stored high-frequency continuous timeseries signals. All admitted ICU patients received intensive monitoring of the vital parameters during ICU stay. Data are stored within the Philips data warehouse (PDW), which is a relational database, ensuring a structured arrangement. The PDW establishes a connection between the data and a unique identifier for efficient management and retrieval. In addition to high-frequency timeseries, other unstructured clinical parameters such as image data and natural language data from the EMR are stored.

### On-premises data warehouse

The ICU monitors are connected to the on-premises PDW, enabling direct data extraction. This structuring is desirable for both clinical utilisation and research purposes. While PDW efficiently handles various data types, including strings, binary large objects, and numbers, streamlined data extraction for complex queries present certain challenges. Moreover, traditional data warehousing structures may not be optimised for computational purposes. To address these challenges, we have utilised cloud environments as a solution and to enhance overall capabilities. High-frequency monitor data from the PDW is transited into the cloud environment. The PDW subsequently follows a data retention policy where information is disposed after a period of two years. Data from the EMR, such as demographic information, medical history, and lab results are directly transited from the EMR into the cloud environment.

### Cloud environment

A cloud environment is a versatile service facilitating data storage, analysis, business intelligence and cloud computation, operating on remote data servers. Managed by third-party providers, these virtual servers offer secure access via either public or private network connections. At Amsterdam UMC, we use Snowflake Inc (Bozeman, Montana, US) with a private network, bypassing the public internet to minimise the risk of unauthorised access or data interception.

This cloud environment allows end-users to control the data flow and perform custom data extractions using SQL-like syntax. In addition to patient data, the cloud environment also facilitates the storage of institutional data, thus enabling analysis of a healthcare institution's administrative and financial information.

The data is stored within the cloud environment as a raw full copy, including noise and artifacts. The use of streaming data pipelines permits the near-instantaneous transfer of this data to the cloud environment, enabling real-time monitoring capabilities. In combination with the natural language data from the EMR clinicians and researchers are able to conduct targeted searches for specific events within the dataset. This allows for the identification of specific events, from which time-related data can be extracted and used in data queries. Integration of these time elements enhances the analysis of trends, correlations, and patterns within the patient data. Furthermore, there is a central extraction point to control all data operations such as uploading, processing and querying within one secure environment, reducing the need for data transfers and data copies across systems. Data is not stored in this extraction point, but serves only as a temporary location to perform these operations. By minimizing unnecessary data movement, the risk of data leakage is reduced.

### Evaluation

The feasibility of the pipeline was evaluated across two domains: clinical practice and research. Within the healthcare domain, we highlighted the utilisation of data in cloud environments to gain insight in the quality of healthcare and explained the role of these data in understanding current protocols and suggesting improvements.

To evaluate the applicability of cloud environments for research, we performed a data query of patients who suffered from a cardiac arrest during their ICU stay. These queried data could be used to develop an algorithm to predict cardiac arrest. To extract these data, we performed a keyword search in the natural language data of the EMR to select patients who suffered from a cardiac arrest in the ICU. We used the following keywords: “*IHCA” (*in hospital cardiac arrest*)*, “*arrest”*, “*botboor”* (bone drill), “*CPR”* (cardiopulmonary resuscitation), “*ROSC”* (return of spontaneous circulation), “*VF”* (ventricle fibrillation), and “*PEA”* (pulseless electrical activity). We subsequently excluded cases where cardiac arrest occurred outside the ICU using the keyword: “*OHCA”* (out of hospital cardiac arrest). Patient identifiers were collected with comprehensive inclusion and exclusion word combinations and added to the list upon detecting one or more keyword hits in the EMR. To validate this method, we manually screened daily ICU admission from December 2022 to June 2023 for cardiac arrest cases and compared them to the keyword-based patient selection list from the corresponding period.

### Role of funders

The funder of the study had no role in study design, data collection, statistical analysis, results interpretation, or writing of the report.

## Results

### Summary of available data

**Fig. 2:**
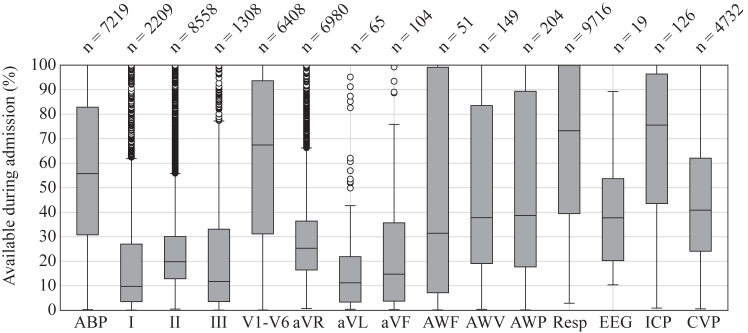
**Box plots of available ICU monitor data for each individual signal, calculated as a percentage of the total ICU stay time and averaged across the patient population for which the signal was available in the dataset. The number of patients contributing to each signal is denoted as ‘n’. ABP, Arterial blood pressure; ECG-leads: I, II, III, V1–V6, aVR, aVL and aVF; AWF, Airway Flow; AWV, Airway Volume; AWP, Airway Pressure; Resp, Respiratory Impedance; EEG,** E**lectroencephalogram; ICP, Intracranial Pressure; CVP, Central Venous Pressure**.

The ICU of the Amsterdam UMC, location AMC, equipped with 50 beds, managed a total of 9716 ICU admissions from June 2021 until June 2023. All monitored high-frequency timeseries data stored in the cloud environment, are presented in Fig. 2. This visualization provides insight in the amount of data that is collected per admission per signal, and the number of patients for which the signal is recorded.

Table 1 shows the distribution of all available data per signal, in total and per individual patient admission, currently stored in the cloud environment. The dataset consists of rows representing 5.12 s of timeseries data, distinguished by distinct wave identifiers corresponding to monitored signals within individual patient admissions. For each wave identifier, we counted the number of rows connected to this identifier and were able to determine the distribution per signal. Wave identifiers with less than 60 min of data (or less than 703 rows) were excluded from analysis. In most of these cases, no physiological signal was recorded, suggesting that the sensor was already active but not connected to the patient. In the results, arterial blood pressure (ABP) was the most recorded signal, with a total recording time of 343,489 h with a median duration of 17 h (IQR: 6.9–49.8) and mean duration of 51 h (SD: 98.8) per patient, and only 0.0028% of data excluded. Electrocardiogram (ECG) signals across various leads showed median durations ranging from 7.5 to 18.1 h and mean durations from 18.4 to 30.4 h, with exclusion rates ranging from 0.0047% to 0.0077%. Additionally, signals such as airway flow (AWF) and airway volume (AWV) exhibited median durations of 11.1 h and 60.6 h, respectively, and mean durations of 45.1 h and 114.9 h.

**Table 1:** Overview of available data in the ICU, collected from June 2021 until March 2023.

Signal	Total hours	Median (Q1—Q3)	Mean (SD)	Excluded (%)
ABP	343,489	17.0 (6.9–49.8)	51.4 (98.8)	0.0028
ECG				
I	62,319	7.5 (2.5–30.5)	30.4 (57.5)	0.0047
II	182,153	7.7 (3.3–20.0)	23.0 (51.8)	0.0062
III	59,941	13.8 (3.3–52.9)	49.7 (88.6)	0.0029
V1	103,112	10.8 (5.2–20.3)	18.4 (26.1)	0.0077
V2	167,339	17.5 (9.1–30.8)	28.3 (40.6)	0.0050
V3	165,990	17.5 (9.3–30.7)	28.2 (40.2)	0.0050
V4	165,034	17.5 (9.0–30.4)	28.1 (40.3)	0.0051
V5	166,289	17.3 (9.1–30.6)	28.1 (39.9)	0.0051
V6	165,576	17.6 (9.2–30.6)	28.1 (39.9)	0.0051
aVR	135,198	7.7 (3.4–19.9)	20.9 (39.5)	0.0068
aVL	1475	15.4 (6.3–32.0)	25.4 (27.6)	0.0056
aVF	2937	18.1 (7.3–38.9)	29.1 (30.5)	0.0049
AWF	2298	11.1 (2.8–52.9)	45.1 (65.3)	0.0032
AWV	16,091	60.6 (15.9–136.1)	114.9 (154.7)	0.0012
AWP	17,681	45.4 (10.8–123.4)	93.1 (130.11)	0.0015
Resp	632,813	19.6 (7.1–60.8)	70.3 (163.5)	0.0020
EEG	3009	154.5 (93.6–177.1)	167.2 (126.8)	0.009
ICP	10,908	47.6 (18.2–158.6)	92.4 (108.2)	0.0015
CVP	158,930	14.1 (6.44–35.0)	35.9 (65.1)	0.0040

The total hours represent the total amount of data per signal. The median and mean are averaged over the number of patients for which the signal was recorded. The percentage of excluded signal was presented in the last column. ABP, arterial blood pressure; PLETH, plethysmogram; ECG, electrocardiography; AWF, airway flow; AWV, airway volume; Resp, respiratory impedance, EEG, electroencephalography; ICP, intracranial pressure; CVP, central venous pressure.

The unique patient identifier, corresponding to one patient admission, enables users to aggregate all signals recorded during that admission. These identifiers enable data queries of individual patients and selection of a patient population for research purposes. Due to the high computational power and scalability of cloud servers, large data queries and other high data volumes are handled efficiently.

### Healthcare evaluation

Furthermore, the obtained data allow for retrospective analysis of the quality of the existing protocols in the ICU. For instance, the intracranial pressure (ICP) is often monitored in traumatic brain injury patients and should not rise above a preset value, commonly around 20–22 mmHg.^11^^,^^12^ The collected data can be used retrospectively to compute the time a patient was exposed to elevated ICP levels and thereby the adequacy of interventions can be assessed. Subsequently, comparison between length of elevated ICP levels and patient outcomes, the protocol can be assessed on its effectiveness and improved if necessary.

From a logistical perspective, cloud environments offer a pathway for assessing ICU bed occupancy. Such an analysis can yield valuable insights into staff workload and establish potential correlations in bed occupation across different medical centres within the region. Furthermore, this approach can aid in determining optimal bed allocation for incoming ICU patients.

### Research evaluation

The manual screening of cardiac arrests in the ICU over the period between December 2022 and June 2023, resulted in 25 recorded cardiac arrests in the ICU and is considered as the ground truth. However, applying keyword search in the EMR over the same period resulted in more patient identifiers, see Table 2. This overview also includes the number of overlapping patient identifiers with the manual search per keyword. Combining all keywords and excluding patients with *‘OHCA’* resulted in 665 unique patient identifiers, including the 25 manually screened identifiers. However, this methodology is insufficient for exclusive patient population selection, as it excludes patients with cardiac arrest in the ICU with an initial out of hospital cardiac arrest (OHCA). Additionally, since medical history is often included in the medical notes, created during current admission, selected patient identifiers associated with a previous history of cardiac arrest are potentially false positive.

**Table 2:** A summarised overview of patient identifiers retrieved from natural language data from the period December 2022 to June 2023.

Keyword	Total number of patient identifiers (n)	Number of patient identifiers after exclusion ‘OHCA’ (n)	Matching number of patient identifiers (n)
IHCA	49	43	14
Arrest	172	152	13
Botboor	5	4	1
CPR	30	27	5
ROSC	121	81	19
VF	117	92	11
PEA	60	39	14
OHCA	103	0	8

The initial count encompasses all identifiers using the corresponding keyword. After excluding identifiers containing ‘OHCA’ in the data, the subsequent count is specified in the second column. The third column denotes the count of identifiers corresponding to the 25 manually screened ICU cardiac arrest cases. Matching was conducted prior to excluding ‘OHCA’ identifiers from the dataset. IHCA, in hospital cardiac arrest; Botboor, bone drill; CPR, cardiopulmonary resuscitation; ROSC, Return of spontaneous circulation; VF, ventricular fibrillation; PEA, pulseless electrical activity; OHCA, out of hospital cardiac arrest.

### Privacy and safety

The monitoring and collection of the patients’ vital parameters is standard clinical practice. Conventionally, the data of all patients admitted to the ICU is stored in the cloud environment and available for clinical and research purposes. Before any data is used for research purposes, patients or their legal representative will be informed on the potential use of their data in research initiatives. An opt out procedure is in place, allowing patients or their representatives to object to the use of their data at any time, both during and after ICU admission. Currently, researchers are responsible for manually excluding these patients from analyses.

To further ensure patient privacy and compliance with relevant regulations, no identifiable personal information is stored in the cloud. Moreover, Amsterdam UMC conducted a risk analysis and established a detailed agreement with Snowflake outlining measures for General Data Protection Regulation (GDPR) compliance, breach liabilities, third-party damage claims handling, protocols for audit reporting and timely leak notifications.

In Snowflake, a data governance layer with numerous tools is deployed to preserve the security, availability, and quality of the data. An important feature is role-based access control: different roles can be assigned with specific authorities and are controlled and centralised at the IT department of the Amsterdam UMC. These roles can be designed for specific tasks using filters for anonymization, data masking, and encryption. These roles can only be assigned to users who are affiliated with the Amsterdam UMC and are exclusively accessible through multifactor authentication. An additional safeguard to ensure data security entails implementation of dual encryption keys. These keys are in possession of the Amsterdam UMC and the cloud service provider and must be unlocked simultaneously to enable data transmission between both entities.

## Discussion

The aim of this paper was to outline a method for optimizing the storage and usage of data in data-dense environments such as the ICU for both clinical and scientific applications. The proposed data infrastructure, implemented at the Amsterdam UMC, effectively organises, stores, and manages large amounts of data, enabling retrospective analyses that may contribute to better understanding of a clinically relevant research question or an individual clinical setting. Beyond patient data, available institutional data enable assessment of the quality of healthcare institutions, including the evaluation of patient outcomes, adherence to clinical protocols, infection rates, medication errors, and readmission rates. Additionally, cloud environments enable the combination of large amounts of data with the computational power, the high uptime, and the streaming data pipelines. The reliability and availability of these services provide the opportunity for real-time data driven AI applications in the ICU setting, including predictive algorithms that could potentially set off an alarm in case a clinical event is predicted and might facilitate prevention of these events by proactive treatment. Therefore, it is essential to minimise latency, which is a customizable feature, to ensure immediate data processing and transmission. However, the implementation of such technologies requires further research and validation.

### Research applications

Data extraction from the cloud environment, as illustrated by our research focusing on cardiac arrest prediction in the ICU, presents challenges. Researchers are constrained to prospective manual screening of all patients in the ICU, since there is no automated event registration incorporated in the cloud environment and keyword search resulted in excessive false positives. The current proof of concept will benefit from an accurate event registration by enabling research on very specific events and patient populations. To improve the accuracy of patient selection, we recommend enhancing or automating the registration of critical events in the ICU or extending this approach throughout a hospital stay. In the case of cardiac arrest, this could involve monitoring the administration of adrenalin at a dose of 1 mg, as these high doses are exclusively provided for treatment during resuscitation. However, because of the urgent nature of cardiac arrest, drug administration is often not prioritised, and event registration is not initially deployed in cloud environments, serving merely as a platform for data storage and cloud computation purposes. Therefore, additional event registration must be implemented to optimise the use of cloud environments for research and clinical purposes.

Researchers are not exclusively assigned to the use of keyword search to obtain the data. Data in the cloud environment can be selected based on multiple characteristics such as the type of signal and timestamp. However, such a broad data query would obtain data irrelevant to our scope. Therefore, the data manager or the Institutional Review Board should not approve such queries without a thorough research plan.

The research purpose in this example aligns with research focusing on prevention of diseases with the available data in the ICU. The available monitoring data allow training and validation of machine learning based algorithms followed by real-time deployment in the ICU. Real-time application of these algorithms involves processing large volumes of data, which demands significant computational resources. Cloud environments support this by providing the necessary computational power and enabling scalability, ensuring that resources are allocated as needed, thereby optimizing costs. The vast amount of data combined with the computational power of the cloud environments enable the application of predictive algorithms using artificial intelligence. A recent study by Sun et al. have demonstrated integrated systems for multimodal data acquisition and analysis in the ICU, which shows the potential for such data-driven prediction applications in clinical settings.^13^ However, the practicality of employing these models requires further investigation.

### Safety and security

Currently, most hospitals rely on local infrastructures with on-premise servers and have therefore more control over their local networks and can adopt a preferred security strategy. The introduction of cloud environments is a new development and therefore brings uncertainty in terms of data security and transparency.^14^ Storage of patient data ‘in the cloud’ often requires the involvement of a third party which means that the hospital has less control over the data storage process. However, all layers in the pipeline are fully designed to meet the specific requirements of the Amsterdam UMC. While third parties provide the tools for a secure environment, the institution retains control over the design and customization of the system. In this paper, the third-party provider was selected based on its strong security measures, including private networking and role-based access controls. These features make this one of the safest approaches for storing large amounts of data. Additionally, third party providers specialise in data security and high-performance cloud storage and computing. They typically have more resources than the Amsterdam UMC, not just in terms of budget but also in the capacity, availability and reliability of servers and its management, ensuring a higher level of security and performance. Agreements are established regarding data storage location, accessibility, and sharing.

### Legal reflections on cloud transitioning

Cloud-based healthcare and research applications require careful consideration of, and compliance with, complex (and currently evolving) legal, and regulatory frameworks.^15^ These include national and international data protection regulations, such as the GDPR in the European Union^16^ and similar legislation in many other jurisdictions.^15^ The GDPR, for example, governs processing of personal data, including the collection, storage, and use of health-related information. In addition, the forthcoming EU Artificial Intelligence (AI) Act establishes a legal framework governing the development and deployment of AI systems, including stringent requirements for certain applications deemed to be “high-risk” (a category that will include certain healthcare applications).^17^

Amsterdam UMC has established protocols to ensure the secure handling of patient data, restricting access to authorised medical staff and maintaining accountability through logged and audited activities. The hosting servers are physically located in The Netherlands, ensuring compliance with GDPR data residency requirements in the EU. Snowflake was chosen based on a thorough evaluation of several criteria, including its robust security measures, scalability, flexibility, and ability to align with the institute's strict data management protocols.

To protect patient privacy, the anonymization and de-identification of data are carried out following standardised procedures that ensure compliance with GDPR regulations. This process involves replacing identifiable information with coded representations, ensuring that all shared data is anonymised by default. The keys for pseudonymization are securely managed to maintain strict control over re-identification, permitted only under authorised circumstances.

Before formalizing agreements with Snowflake, all legal aspects were thoroughly reviewed and, if required, revised by the legal department and privacy officers. A comprehensive risk analysis ensured that the architecture complies with the GDPR requirements, legally binding Snowflake to Amsterdam UMC's strict standards.

Local laws and ethical codes regulating the provision of healthcare further complicate the landscape. For example, in the Netherlands, 7:453 of the civil code states that “the health-care provider must act as a good health-care provider,” which creates an obligation for the health-care provider to provide good care.^18^ By extension, any changes to the healthcare system should ultimately benefit the welfare of patients in one way or another. As the transition to cloud-based environments is considered, whether this transition contributes to good care, should be assessed.

In addition to legal compliance, cloud-based clinical decision-making require broader ethical considerations, including implications for patient privacy, autonomy, and informed consent. Additional research (e.g., into the extent to which patients are aware of how data about them is used, protected, and with whom it is shared) and best practice development in this area is required (e.g., obtaining informed consent in the age of cloud-based computing and artificial intelligence). These legal and ethical complexities necessitate a collaborative approach (e.g., between institutions, healthcare providers, and researchers) that should ultimately balance patient welfare, privacy, and informed consent with innovation and the tremendous potential of cloud-based clinical decision-making to improve patient care.

### Environmental impact

Cloud environments offer optimal data storage and the potential for reduced environmental impact through approaches such as data mutation and time travel. For example, data mutation involves the creation of new data instances instead of modifying existing data directly, conserving space, and maintaining data integrity by minimizing duplication.^19^ Time travel complements this by enabling users to access historical data snapshots at different points in time, facilitating historical analysis.^20^ However, the need to maintain readily available medical data may lead healthcare institutions to combine cloud-based storage with local backups, a dual storage approach that could result in higher energy consumption due to use of redundant systems.

In summary, this paper demonstrates a successful proof of concept for utilising cloud environment solutions for the efficient storage and management of ICU data, particularly high-frequency monitor data. However, its potential should be further expanded for optimal use of the cloud environment. Expanding the capabilities to include automated event registration would optimise retrospective analysis on the data, enhancing the utility of the data for both research and clinical evaluation. This expansion could lead to real-time data driven clinical decision-making applications, which is supported by cloud environments. Furthermore, institutions are encouraged to perform a thorough risk analysis before finalizing agreements with the cloud environment provider. In the future, extending the use of cloud systems to multiple departments and healthcare nationwide could enhance our understanding of individual patients, patient populations and healthcare overall.

## Contributors

SHN, APV and BJPS conceptually designed the study. SHN, BJPS and FCB contributed to the design and of the methodology and were involved in implementing the data analysis. SHN, BJPS and FCB directly accessed to the underlying data reported in the manuscript. DPV contributed to the clinical context of the pipeline and the manuscript. APV and BJPS provided oversight and supervision. All authors contributed to the writing of the manuscript and gave their final approval. In addition, the corresponding author attests that all listed authors meet authorship criteria.

## Data sharing statement

Data is unavailable from this study.

## Declaration of interests

APJV declares consulting fees, speakers fees, and an unrestricted research grant paid from Edwards LifeSciences to the institute. The remaining authors have nothing to declare.
